# Safety and effectiveness of eribulin in Japanese patients with soft tissue sarcoma including rare subtypes: a post-marketing observational study

**DOI:** 10.1186/s12885-022-09527-y

**Published:** 2022-05-11

**Authors:** Akira Kawai, Hiroyuki Narahara, Shunji Takahashi, Tomoki Nakamura, Hiroshi Kobayashi, Yasunori Megumi, Toshiyuki Matsuoka, Eisuke Kobayashi

**Affiliations:** 1 Department of Musculoskeletal Oncology and Rehabilitation Medicine, National Cancer Center Hospital, Rare Cancer Center, National Cancer Center Hospital, 5-1-1 Tsukiji, Chuo-ku, Tokyo, 104-0045 Japan; 2 Rare Cancer Center, National Cancer Center, Tokyo, Japan; 3 Department of Medical Oncology, Hyogo Prefectural Nishinomiya Hospital, Nishinomiya, Japan; 4 Department of Medical Oncology, The Cancer Institute Hospital of Japanese Foundation for Cancer Research, Tokyo, Japan; 5 Department of Orthopedic Surgery, Mie University Graduate School of Medicine, Tsu, Japan; 6 Department of Orthopedic Surgery, Faculty of Medicine, The University of Tokyo, Tokyo, Japan; 7 Clinical Planning and Development Department, Medical Headquarters, Eisai Co., Ltd., Tokyo, Japan

**Keywords:** Eribulin, Japan, Post-marketing study, Soft tissue sarcoma, Overall survival

## Abstract

**Background:**

Soft tissue sarcomas (STSs) are a heterogeneous group of cancers with over 100 described subtypes. While these cancers are infrequent, the prognosis is quite poor, particularly for those with stage IV metastatic disease. Patients for whom curative resection is difficult or those with recurrent metastatic disease are treated with chemotherapy, although the options are very limited. Eribulin is an approved treatment of all STS subtypes in Japan. Efficacy and safety data for the treatment of rare STS subtypes other than liposarcoma and leiomyosarcoma (L-type sarcomas) are limited. This nationwide, multicenter, prospective, post-marketing observational study was conducted to assess the real-world effectiveness and safety of eribulin in Japanese patients with STS.

**Methods:**

Patients with all types of STS and who consented to eribulin treatment were eligible to participate. The observation period was 1 year, starting at treatment initiation, and clinical outcomes were followed up for 2 years after initiating treatment. The primary endpoint was overall survival (OS). Additional outcomes included time-to-treatment failure (TTF), objective response rate (ORR), disease control rate (DCR), and safety. ORR and DCR were evaluated using imaging findings. Effectiveness results were analyzed both for all patients and by STS subtype.

**Results:**

A total of 256 patients were enrolled; 252 and 254 were included in the effectiveness and safety analysis set, respectively. Most patients (83.1%) received an initial eribulin dose of 1.4 mg/m^2^ (standard dose). Respective median OS (95% confidence interval [CI]) was 10.8 (8.5–13.1), 13.8 (10.1–22.3) and 6.5 (5.7–11.1) months for all, L-type, and non-L-type subtypes. The respective median TTF (95% CI) was 2.5 (2.1–2.8), 2.8 (2.3–3.7), and 2.2 (1.6–2.6) months. The ORR and DCR were 8.1 and 42.6%, respectively. Adverse drug reactions (ADRs) and serious ADRs were reported for 83.5 and 18.9% of patients, respectively. The main ADRs were associated with myelosuppression. No significant difference was observed in the incidence of ADRs for patients ≥65 versus <65 years old.

**Conclusions:**

Eribulin demonstrated effectiveness and a manageable safety profile for patients with STS, although the effectiveness of eribulin was not demonstrated for some non-L-type subtypes.

**Trial registration:**

NCT03058406 (ClinicalTrials.gov).

**Supplementary Information:**

The online version contains supplementary material available at 10.1186/s12885-022-09527-y.

## Background

Soft tissue sarcomas (STSs) are an infrequent and heterogeneous group of cancers with over 100 described histological subtypes [1]. The most commonly occurring histological subtypes are leiomyosarcomas, liposarcomas, undifferentiated pleomorphic sarcoma, malignant peripheral nerve sheath tumor, and synovial sarcoma [2]. A recent survey reported that there were approximately 4000 patients with STS in Japan [3] and that there are approximately 2000 deaths from STS annually [4]. The 5-year survival rate for STS is 65% [5]. In the metastatic setting, patients with STS have an extremely poor prognosis, with a survival time of about 1 year after treatment [6–8]; prognosis is especially poor for stage IV patients with metastasis.

The standard first-line treatment for localized STS is surgery, radiation, or a combination of both [9]. Curative resection of the primary tumor is the mainstay of treatment both in Japan and overseas. Chemotherapy is used to treat patients for whom curative resection is difficult or who have recurrent or metastatic disease. The standard chemotherapy regimen for STS is either single-agent doxorubicin or an anthracycline in addition to combination therapy centered on doxorubicin and ifosfamide [10]. The objective response rate (ORR) for these therapies ranges between 5 and 25%, and the median progression-free survival (PFS) and median overall survival (OS) are 2–7 months and 10–14 months, respectively [11–13]. Pazopanib was approved in 2012 as a second-line treatment option for STS after initial chemotherapy both in Japan and overseas. The median PFS and median OS for patients treated with pazopanib were 4.6 and 12.5 months, respectively; the overall response was 6% [14]. Non-pazopanib treatment options include trabectedin monotherapy, gemcitabine-docetaxel combination therapy, dacarbazine monotherapy, and dacarbazine-gemcitabine combination therapy. These options are used in patients who have either failed or had difficulty continuing initial chemotherapy and, aside from trabectedin monotherapy, are only available outside Japan or are not covered by Japan’s universal health insurance system. However, in some cases, unapproved therapies (e.g., gemcitabine-docetaxel combination) can be prescribed by the treating physicians in accordance with Japanese guidelines [10]. The reported ORR for non-pazopanib therapies ranges from 5 to 15%, and median PFS and OS range from 2 to 6 months and 8 to 18 months, respectively [15–17]. In Japan, patients who have failed doxorubicin, ifosfamide, or pazopanib treatment, or who have difficulty continuing treatment because of adverse reactions (such as cardiotoxicity, nephrotoxicity, and hepatotoxicity) have no other treatment options, making chemotherapy options for malignant STS very limited. Given the above, there is a substantial unmet need for new therapies, particularly in Japan.

Eribulin mesylate, hereafter referred to as eribulin, is a fully synthetic macrocyclic ketone analog of the marine natural product halichondrin B that inhibits microtubule dynamics and has antitumor effects by mitotic blockade [18]. Japan is the only country that has approved eribulin treatment for all STS subtypes [19]. This approval was based on the phase 2 study of eribulin in patients with advanced/metastatic STS (NCT01458249), which was conducted in Japan, and the phase 3 study of eribulin in patients with advanced liposarcoma/leiomyosarcoma (NCT01327885) [20–22]. In the United States, eribulin is only approved for liposarcoma subtypes [21]. Efficacy and safety data for eribulin treatment of rare STS subtypes other than liposarcoma and leiomyosarcoma (L-type sarcomas) are limited. The objective of this post-marketing observational study was to assess the real-world effectiveness and safety of eribulin in Japanese patients with STS stratified by subtype (L-type and non-L-type) who were followed up for 2 years.

## Methods

### Patients

Japanese patients with all types of STS (L-type and non-L-type) who met the criteria for STS according to the 2013 World Health Organization classification [23] and who consented to eribulin treatment were eligible for inclusion in this study. Key exclusion criteria included severe myelosuppression, a history of hypersensitivity to eribulin, and pregnancy or having the potential to become pregnant.

### Study design and treatment

The detailed methodology used in this study has already been published [24]. Briefly, this was a nationwide, multicenter, prospective, post-marketing observational study conducted in Japan between February 2016 and January 2019. Patients were enrolled in the study across 102 institutes where they were receiving eribulin treatment. Eribulin was administered intravenously at a dose of 1.4 mg/m^2^ (2–5 min) on days 1 and 8 of each 3-week treatment cycle. Dose adjustments were allowed at the discretion of the treating physician and in accordance with the eribulin prescribing information [25].

The observation period was 1 year, which started at treatment initiation; clinical outcomes were followed up for up to 2 years after treatment initiation. In addition to collecting baseline data prior to treatment initiation, the treating physician completed questionnaires at 3 months, 1 year, and 2 years after initiation of eribulin treatment. Data collected included patient characteristics, clinicopathological data and treatment history at baseline, eribulin administration status, concomitant drugs, diagnostic imaging, adverse events (AEs), adverse drug reactions (ADRs), and clinical outcome.

The study was conducted in accordance with the Declaration of Helsinki and Good Post-Marketing Study Practice. Approval from Clinical Review and Approval Board at Eisai Co., Ltd., and the ethics committee or institutional review board at each study institute was obtained prior to study initiation. Because this was an observational study based on Good Post-Marketing Study Practice, formal informed consent was waived by Clinical Review and Approval Board at Eisai Co., Ltd.; however, all patients consented to eribulin treatment. This study was registered on ClinicalTrials.gov (NCT03058406).

### Effectiveness and safety

The primary endpoint was OS, defined as the time from initiation of eribulin treatment to death from any cause. Time-to-treatment failure (TTF), ORR, disease control rate (DCR), and safety were also evaluated. TTF was defined as the time from the first dose of eribulin until the date of treatment discontinuation from any cause; it was censored at the date of last follow-up for surviving patients remaining on treatment. Imaging findings were used to evaluate ORR and DCR. ORR was defined as complete response (CR) + partial response (PR) and DCR as CR + PR + stable disease (SD). ORR was determined by the responsible physicians at each institution using the Response Evaluation Criteria in Solid Tumours guideline (version 1.1). The timing of diagnostic imaging for these time-to-event outcomes was based on the treating physician’s discretion. AEs were assessed according to the Common Terminology Criteria for Adverse Events (Japanese version 4.0). AEs for which a causal relationship to eribulin could not be ruled out were regarded as ADRs. Effectiveness results were analyzed for all patients and by STS subtype.

### Statistical analysis

Sample size calculations were performed considering that a sufficiently large sample would be necessary to detect Grade ≥ 3 infections associated with myelosuppression. The sample size was set to evaluate safety of eribulin treatment at the recommendation of Pharmaceuticals and Medical Devices Agency. In the Japanese phase 2 study of patients with STS [20], infections and infectious pleural effusion occurred in 2% of patients, and these events were less frequent among Grade ≥3 infections. Based on this, we calculated a 95% probability that at least one event, which was expected to occur in 2% of the population, could be detected in 150 patients. With an assumed withdrawal rate of 5%, the planned sample size was 160 patients. When the required number of patients had been enrolled, patient registration was stopped after consultation with the authorities.

The safety analysis set comprised all patients who received at least one dose of eribulin and had at least one safety assessment. The effectiveness analysis set comprised all patients with a diagnosis of STS. Safety and effectiveness data were evaluated using descriptive statistics; OS and TTF were assessed using the Kaplan–Meier method. Differences in safety measures among subgroups were evaluated using a chi-square test. All statistical analyses were performed using SAS version 9.4 (SAS Institute Inc., Cary, NC, USA).

## Results

### Patients and treatment

**Fig. 1:**
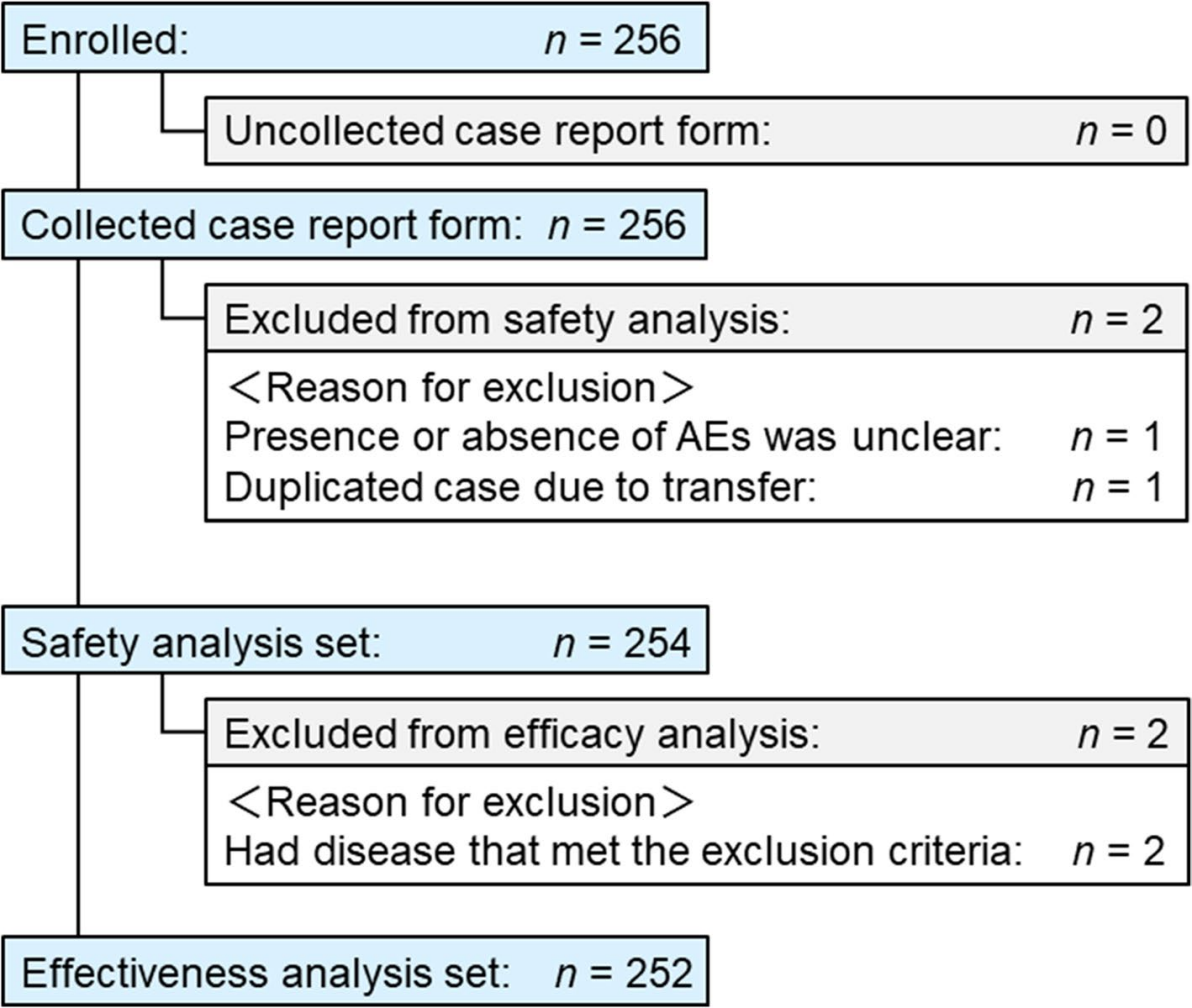
Patient disposition. *AE* adverse event

Patient disposition is shown in Fig. 1. In total, 256 patients were enrolled in the study and 254 were included in the safety analysis set. One patient was excluded because the presence or absence of AEs was unclear. In addition, one patient was excluded because there was a case duplication due to patient transfer. The effectiveness analysis set included 252 patients; two patients were excluded because they did not have a diagnosis of STS.

Baseline patient characteristics, both for the total population and stratified by age (<65 and ≥65 years), are shown in Table 1. The median age was 62 years (range: 17–87 years), and the proportion of patients aged ≥65 years was 42.5%. The median time from the first onset of STS to the start of eribulin treatment was 2.42 years (range: 0.2–29.2 years), and the percentage of patients with an Eastern Cooperative Oncology Group performance status (ECOG PS) of 0 or 1 at the start of eribulin treatment was 83.9%. The most frequent locations of the main target lesions were the retroperitoneum and abdominal cavity (40.6%), viscera (28.0%), and thoracic cavity (22.0%). The median number of chemotherapy regimens administered prior to initiating eribulin treatment was 2.0 (range: 0–11), and the most common previously administered chemotherapy regimens were doxorubicin monotherapy (36.6%), pazopanib (32.3%), and gemcitabine + docetaxel (26.8%). The proportion of patients who received eribulin as first-line treatment was approximately 5.4% higher in patients aged ≥65 years than in patients aged <65 years. Among patients aged ≥65 years, none received eribulin as fifth-line treatment or above.

**Table 1:** Baseline patient characteristics

	<65 years (***n*** = 146)	≥65 years (***n*** = 108)	ALL (***N*** = 254)
Sex, female	78 (53.4)	56 (51.9)	134 (52.8)
Age, years			
Mean ± standard deviation	50.6 ± 11.1	71.2 ± 5.2	59.3 ± 13.6
Median (range)	53 (17–64)	70 (65–87)	62 (17–87)
ECOG PS			
0	56 (38.4)	43 (39.8)	99 (39.0)
1	68 (46.6)	46 (42.6)	114 (44.9)
2	15 (10.3)	13 (12.0)	28 (11.0)
3	6 (4.1)	6 (5.6)	12 (4.7)
4	1 (0.7)	0	1 (0.4)
Time from diagnosis to initiation of eribulin treatment (years) (*n* = 238)			
Mean ± standard deviation	3.57 ± 3.59	4.74 ± 5.54	4.07 ± 4.57
Median (range)	2.28 (0.2–29.2)	2.50 (0.3–28.8)	2.42 (0.2–29.2)
Target lesion (duplicate count)			
Head and neck	6 (4.1)	10 (9.3)	16 (6.3)
Trunk	24 (16.4)	7 (6.5)	31 (12.2)
Thoracic cavity	32 (21.9)	24 (22.2)	56 (22.0)
Retroperitoneum and abdominal cavity	52 (35.6)	51 (47.2)	103 (40.6)
Upper extremities	5 (3.4)	2 (1.9)	7 (2.8)
Lower extremities	22 (15.1)	10 (9.3)	32 (12.6)
Viscera	43 (29.5)	28 (25.9)	71 (28.0)
Genitourinary	3 (2.1)	3 (2.8)	6 (2.4)
Digestive	9 (6.2)	9 (8.3)	18 (7.1)
Gynecologic	17 (11.6)	9 (8.3)	26 (10.2)
Breast	4 (2.7)	0	4 (1.6)
Others	12 (8.2)	10 (9.3)	22 (8.7)
Others	13 (8.9)	11 (10.2)	24 (9.4)
Soft tissue sarcoma subtype			
Leiomyosarcoma	43 (29.5)	30 (27.8)	73 (28.7)
Liposarcoma	39 (26.7)	30 (27.8)	69 (27.2)
Dedifferentiated	19 (13.0)	21 (19.4)	40 (15.7)
Myxoid	10 (6.8)	2 (1.9)	12 (4.7)
Well-differentiated	5 (3.4)	2 (1.9)	7 (2.8)
Pleomorphic	2 (1.4)	1 (0.9)	3 (1.2)
Unknown	3 (2.1)	4 (3.7)	7 (2.8)
Undifferentiated pleomorphic sarcoma	11 (7.5)	8 (7.4)	19 (7.5)
Angiosarcoma	5 (3.4)	9 (8.3)	14 (5.5)
Synovial sarcoma	11 (7.5)	2 (1.9)	13 (5.1)
Rhabdomyosarcoma	7 (4.8)	5 (4.6)	12 (4.7)
Malignant peripheral nerve sheath tumor	4 (2.7)	2 (1.9)	6 (2.4)
Myxofibrosarcoma	0	5 (4.6)	5 (2.0)
Others	26 (17.8)	17 (15.7)	43 (16.9)
Median number of previous chemotherapies (range) (*n* = 251)	2 (0–11)	2 (0–4)	2 (0–11)
Number of previous chemotherapies			
0	7 (4.8)	11 (10.2)	18 (7.1)
1	46 (31.5)	35 (32.4)	81 (31.9)
2	44 (30.1)	29 (26.9)	73 (28.7)
3	24 (16.4)	21 (19.4)	45 (17.7)
4	12 (8.2)	10 (9.3)	22 (8.7)
≥ 5	12 (8.2)	0	12 (4.7)
Unknown	1 (0.7)	2 (1.9)	3 (1.2)
Major previous regimens			
Doxorubicin monotherapy	54 (37.0)	39 (36.1)	93 (36.6)
Pazopanib	45 (30.8)	37 (34.3)	82 (32.3)
Gemcitabine + docetaxel	43 (29.5)	25 (23.1)	68 (26.8)
Doxorubicin + ifosfamide	43 (29.5)	15 (13.9)	58 (22.8)
Trabectedin	28 (19.2)	20 (18.5)	48 (18.9)

*ECOG PS* Eastern Cooperative Oncology Group Performance Status

Eribulin treatment status is shown in Table 2. The proportion of patients who received an initial dose of 1.4 mg/m^2^ (standard dose) of eribulin was 83.1%, and the median number of cycles administered was 3.0 (range: 1–20). When comparing initial doses, the proportion of patients aged ≥65 years who received an initial eribulin dose of 1.4 mg/m^2^ was approximately 17.3% lower than that of patients aged <65 years; the proportion of patients who received a lower initial dose of 1.1 mg/m^2^ was approximately 16.3% higher. The number of cycles administered was similar in patients aged <65 years and those aged ≥65 years.

**Table 2:** Eribulin treatment status

	<65 years (***n*** = 146)	≥65 years (***n*** = 108)	ALL (***N*** = 254)
Initial dose (mg/m^2^)			
1.4	132 (90.4)	79 (73.1)	211 (83.1)
1.1	10 (6.8)	25 (23.1)	35 (13.8)
0.7	0	0	0
Others	4 (2.7)	4 (3.7)	8 (3.1)
Number of cycles			
Mean ± standard deviation	4.8 ± 4.2	5.0 ± 4.2	4.8 ± 4.2
Median (range)	3.0 (1–20)	3.0 (1–18)	3.0 (1–20)
Dose intensity (mg/m^2^/week)			
Mean ± standard deviation	0.71 ± 0.19	0.65 ± 0.21	0.68 ± 0.20
Median (range)	0.71 (0.3–1.0)	0.65 (0.2–0.9)	0.70 (0.2–1.0)
Relative dose intensity			
Mean ± standard deviation	0.76 ± 0.20	0.69 ± 0.22	0.73 ± 0.21
Median (range)	0.76 (0.3–1.0)	0.69 (0.2–1.0)	0.75 (0.2–1.0)

### Effectiveness

OS (primary endpoint) and TTF (secondary endpoint) are shown in Table 3. The median OS (95% confidence interval [CI]) was 10.8 (8.5–13.1) months for all STS subtypes, 12.7 (8.5–20.6) months for leiomyosarcoma, and 20.8 (8.9–not reached) months for liposarcoma. The median (95% CI) OS was 13.8 (10.1–22.3) months for L-type STS subtypes and 6.5 (5.7–11.1) months for non-L-type subtypes. The median (95% CI) TTF for all STS subtypes was 2.5 (2.1–2.8) months. The median (95% CI) TTF was 2.8 (2.3–3.7) months for L-type subtypes and 2.2 (1.6–2.6) months for non-L-subtypes (including undifferentiated pleomorphic sarcoma, angiosarcoma, synovial sarcoma, and others). Both OS and TTF were significantly longer in patients with L-type STS than in patients with non-L-type subtypes (OS: hazard ratio [HR] 2.01, 95% CI 1.47–2.76; TTF: HR 1.60, 95% CI 1.23–2.08, log-rank test).

**Table 3:** Overall survival and time-to-treatment failure after treatment with eribulin

Soft tissue sarcoma subtype^**a**^	***n***	OS median, months (95% CI)	TTF median, months (95% CI)
All	252	10.8 (8.5–13.1)	2.5 (2.1–2.8)
L-type	142	13.8 (10.1–22.3)	2.8 (2.3–3.7)
Non-L-type	110	6.5 (5.7–11.1)	2.2 (1.6–2.6)
Leiomyosarcoma	73	12.7 (8.5–20.6)	2.8 (2.1–3.7)
Liposarcoma	69	20.8 (8.9–not reached)	3.2 (1.7–5.5)
Dedifferentiated	40	16.6 (8.8–not reached)	3.1 (1.7–5.7)
Myxoid	12	27.6 (4.1–27.6)	5.0 (1.0–12.0)
Well-differentiated	7	not reached (5.8–not reached)	9.9 (0.5–not reached)
Pleomorphic	3	7.3 (6.9–8.5)	1.4 (1.4–2.0)
Unknown	7	5.8 (1.8–not reached)	1.5 (0.5–5.8)
Undifferentiated pleomorphic sarcoma	19	8.1 (4.0–16.3)	2.3 (1.4–4.0)
Angiosarcoma	14	12.7 (3.4–16.9)	2.4 (0.7–5.5)
Synovial sarcoma	13	11.7 (4.5–16.0)	3.7 (2.3–4.6)
Rhabdomyosarcoma	12	4.5 (0.8–9.7)	1.2 (0.5–2.8)
Malignant peripheral nerve sheath tumor	6	3.3 (2.0–5.1)	1.5 (0.5–2.3)
Myxofibrosarcoma	5	11.1 (2.3–19.8)	8.1 (0.7–8.4)

*CI* confidence interval, *OS* overall survival, *TTF* time-to-treatment failure

^a^Limited to subtypes with 5 or more patients

The ORR and DCR for eribulin treatment were 8.1 and 42.6%, respectively (Table 4). One patient achieved a best overall response of CR, 18 achieved PR, 81 had SD (47 had SD ≥11 weeks), 129 had progressive disease, and six were not evaluable. Details of the TTF, ORR, and DCR for the STS subtypes that were reported in fewer than five patients are shown in Additional file 1.

**Table 4:** Response rate, objective response rate, and disease control rate

		Response rate, ***n***						ORR (CR + PR)	DCR
Soft tissue sarcoma subtype^**a**^	***n***	CR	PR	SD	SD (≥11 W)	PD	NE	(%)	(%)
All	235	1	18	81	47	129	6	8.1	42.6
L-type	135	1	7	60	37	64	3	5.9	50.4
Non-L-type	100	0	11	21	10	65	3	11.0	32.0
Leiomyosarcoma	71	1	4	30	14	36	0	7.0	49.3
Liposarcoma	64	0	3	30	23	28	3	4.7	51.6
Dedifferentiated	37	0	0	19	15	17	1	0.0	51.4
Myxoid	12	0	3	4	2	3	2	25.0	58.3
Well-differentiated	6	0	0	4	4	2	0	0.0	66.7
Pleomorphic	3	0	0	0	0	3	0	0.0	0.0
Unknown	6	0	0	3	2	3	0	0.0	50.0
Undifferentiated pleomorphic sarcoma	18	0	2	2	1	14	0	11.1	22.2
Angiosarcoma	13	0	2	2	2	9	0	15.4	30.8
Synovial sarcoma	13	0	3	3	1	7	0	23.1	46.2
Rhabdomyosarcoma	11	0	2	0	0	8	1	18.2	18.2
Malignant peripheral nerve sheath tumor	5	0	0	0	0	5	0	0.0	0.0
Myxofibrosarcoma	5	0	1	1	0	2	1	20.0	40.0

*CR* complete response rate, *DCR* disease control rate, *NE* not evaluable, *ORR* objective response rate, *PD* progressive disease, *PR* partial response, *SD* stable disease, *W* weeks

^a^Limited to subtypes with 5 or more patients

### Safety

In total, 83.5% (212/254) and 18.9% (48/254) of patients experienced ADRs and serious ADRs, respectively. ADRs that occurred in ≥3% of patients are listed in Table 5. Neutropenia (60.6%), leukopenia (59.1%), and lymphopenia (15.7%) were the most commonly occurring all grade ADRs. In total, 70.5% (179/254) of patients experienced Grade ≥3 ADRs; neutropenia, leukopenia, and lymphopenia, which occurred in 53.9, 47.6, and 15.0% of patients, respectively, were the most common. Frequent serious ADRs were neutropenia, leukopenia, febrile neutropenia, and anemia in 8.7, 7.9, 2.4, and 1.6% of patients, respectively. No deaths due to ADRs were reported. ADRs leading to discontinuation occurred in 13.4% of patients and included leukopenia and neutropenia (4.3% for each), peripheral neuropathy (1.2%), and febrile neutropenia, lymphopenia, malaise, C-reactive protein increased, and hemoglobin decreased (0.8% for each).

**Table 5:** Adverse drug reactions occurring in ≥3% of patients

	<65 years (***n*** = 146)		≥65 years (***n*** = 108)		ALL (***N*** = 254)	
	All grade	Grade ≥3	All grade	Grade ≥3	All grade	Grade ≥3
Any	120 (82.2)	103 (70.5)	92 (85.2)	76 (70.4)	212 (83.5)	179 (70.5)
Hematological toxicity						
Neutropenia	82 (56.2)	73 (50.0)	72 (66.7)	64 (59.3)	154 (60.6)	137 (53.9)
Leukopenia	84 (57.5)	67 (45.9)	66 (61.1)	54 (50.0)	150 (59.1)	121 (47.6)
Lymphopenia	26 (17.8)	24 (16.4)	14 (13.0)	14 (13.0)	40 (15.7)	38 (15.0)
Anemia	14 (9.6)	10 (6.8)	16 (14.8)	10 (9.3)	30 (11.8)	20 (7.9)
Thrombocytopenia	7 (4.8)	4 (2.7)	2 (1.9)	1 (0.9)	9 (3.5)	5 (2.0)
Febrile neutropenia	3 (2.1)	3 (2.1)	6 (5.6)	6 (5.6)	9 (3.5)	9 (3.5)
Non-hematological toxicity						
Peripheral neuropathy	18 (12.3)	2 (1.4)	12 (11.1)	3 (2.8)	30 (11.8)	5 (2.0)
Stomatitis	5 (3.4)	0	4 (3.7)	1 (1.0)	9 (3.5)	1 (0.4)
Alopecia^a^	7 (4.8)	–	4 (3.7)	–	11 (4.3)	–
Malaise	8 (5.5)	1 (0.7)	5 (4.6)	0	13 (5.1)	1 (0.4)
Pyrexia	7 (4.8)	0	2 (1.9)	0	9 (3.5)	0
Laboratory test abnormalities						
ALT-increased	23 (15.8)	3 (2.1)	10 (9.3)	0	33 (13.0)	3 (1.2)
AST-increased	20 (13.7)	2 (1.4)	12 (11.1)	0	32 (12.6)	2 (0.8)
γ-GTP increased	11 (7.5)	8 (5.5)	2 (1.9)	1 (0.9)	13 (5.1)	9 (3.5)
CRP increased	8 (5.5)	2 (1.4)	9 (8.3)	5 (4.6)	17 (6.7)	7 (2.8)
Hemoglobin decreased	10 (6.8)	4 (2.7)	2 (1.9)	2 (1.9)	12 (4.7)	6 (2.4)

Data are presented as *n* (%). *ALT* alanine aminotransferase, *AST* aspartate aminotransferase, *CRP* C-reactive protein, *GTP* glutamyl transferase

^a^ Grading for alopecia is not available as indicated by ‘-‘

When comparing patients <65 years with those ≥65 years, all grade ADRs occurred in 82.2 and 85.2% of patients in each group, respectively. The respective incidences of Grade ≥ 3 ADRs were 70.5 and 70.4%. There was no numerical difference in either all grade ADRs or Grade ≥3 ADRs between the two groups. ADRs that occurred at more than a 2-fold higher rate in patients aged ≥65 years than those aged <65 years were febrile neutropenia for all grade ADRs, and febrile neutropenia, peripheral neuropathy, and C-reactive protein increased for ADRs of Grade ≥3.

The incidence rate of Grade ≥3 ADRs among patients who were aged ≥75 years (*n* = 26) was 80.8%. Statistical analysis revealed no significant difference between the incidence of Grade ≥3 ADRs in patients ≥75 years of age versus those <65 years of age (70.5%, 103/146 patients) or ≥65 to <75 years of age (67.1%, 55/82 patients) (*P* = 0.4106, chi-square test).

## Discussion

To date, this is the largest study to report OS results for patients with STS, including rare subtypes, who were treated with eribulin in a real-world clinical setting. The interim results of the present study were previously published [24]. As noted, treatment options for rare subtypes of STS are limited. The available efficacy data for STSs are focused on L-type sarcomas, and in the United States and Europe, eribulin is only approved for the treatment of malignant liposarcoma [26, 27]. The OS data for both L-type and non-L-type STS obtained from the present study are unprecedented, providing valuable information for physicians treating patients with STS.

In preclinical studies, eribulin demonstrated potent cytotoxicity in a panel of 24 cell lines [28] and six human STS cell lines (two leiomyosarcoma lines, and one each of liposarcoma, Ewing’s sarcoma, synovial sarcoma, and fibrosarcoma lines) [29], as well as antitumor activity in xenograft models using human STS cell lines (one each of leiomyosarcoma, liposarcoma, Ewing’s sarcoma, and fibrosarcoma) [29]. Additionally, eribulin demonstrated in vitro activity in a primary culture of undifferentiated pleomorphic sarcoma tumor cells; treatment resulted in upregulation of epithelial mesenchymal transition-related genes and genes involved in chemoresistance and downregulation of epithelial markers [30]. The expression of cell cycle arrest-related and pro-apoptotic-related proteins was also observed with in vitro treatment. The safety and efficacy of eribulin for treating liposarcoma has been reported in a phase 3 study [22] and for treating other subtypes in two phase 2 studies, including one conducted in Japan [20, 31]. Together, preclinical and clinical data indicate that eribulin may be effective for many STS subtypes.

The present study reported a median OS of 10.8 months, which is somewhat lower than the 13.2 months reported in a phase 2 study [20] and the 13.5 months reported in the phase 3 study [22]. A possible explanation of the lower OS reported in the present study may be that our study had a higher percentage of patients with non-L-type STS than the previously reported phase 2 and phase 3 studies, as OS is higher for patients with L-type versus non-L-type STS [20, 32]. Along this line, the present study reported that the OS was the longest for patients with liposarcoma (20.8 months) and the shortest for those with rhabdomyosarcoma or malignant peripheral nerve sheath tumor (4.5 and 3.3 months, respectively). TTF was the longest for patients with myxofibrosarcoma (8.1 months) and the shortest for patients with rhabdomyosarcoma or malignant peripheral nerve sheath tumor (1.2 and 1.5 months, respectively).

Currently, cytotoxic chemotherapy is the mainstay of treatment for metastatic STS [33]. The GeDDiS trial conducted a head-to-head comparison of doxorubicin versus gemcitabine/docetaxel and found nearly identical OS, PFS, and ORR, even in patients with leiomyosarcoma, uterine leiomyosarcoma, or undifferentiated pleomorphic sarcoma [34]. There is evidence that specific STS subtypes behave differently, indicating that histology-directed therapy may benefit specific subtypes.

Synovial sarcomas are quite sensitive to chemotherapy, particularly with alkylating agents [33], while angiosarcomas are particularly sensitive to taxanes, such as paclitaxel [9, 35]. An analysis of eleven trials of first-line anthracycline-based chemotherapy in angiosarcoma and the non-randomized European Organisation for Research and Treatment of Cancer (EORTC) trial reported a median OS (*n* = 108) of 9.9 months (95% CI 8.3–12.3), which was not significantly different from the median OS of other STS subtypes [36]. In a retrospective study of patients with metastatic angiosarcomas, the median OS of doxorubicin-containing chemotherapy was 11.0 months (*n* = 70) [37]. For weekly paclitaxel, the reported median OS was 8 months (*n* = 30) [35] and 10.3 months (*n* = 75) [38] in two trials of metastatic angiosarcoma, and 13.1 months (*n* = 47) in another retrospective study [37]. For pazopanib, the median OS in a second-line study was 3.0 months (*n* = 9) [39]. Directly comparing the results of these and the present study is difficult; however, the median OS here was comparable with the median OS of anthracycline-based chemotherapy or doxorubicin-containing chemotherapy and weekly paclitaxel.

In a retrospective study of undifferentiated pleomorphic sarcoma, the median OS with trabectedin was 8.4 months (*n* = 10) [40]. Nakamura et al. reported [41] that the median OS of eribulin in undifferentiated pleomorphic sarcoma (*n* = 13) was “not reached”. Although the median OS of undifferentiated pleomorphic sarcoma in this study (8.1 months) was not as high as that reported by Nakamura et al. [41], it was similar to that of trabectedin [40]. For synovial sarcoma, the median OS was reported to be 15.9 months (*n* = 120) based on the results of seven trials of first-line chemotherapy with either doxorubicin or epirubicin [42]. In a retrospective study of trabectedin, the median OS was 19.3 months (*n* = 18) [40]. In second-line studies, the median OS was “not reached” (*n* = 3) for pazopanib and 3.0 months (*n* = 6) for gemcitabine/docetaxel combination [39].

Schöffski et al. [31] reported that the 6-month survival rates with eribulin were 71.1% (95% CI 43.7–86.8%) for synovial sarcoma (*n* = 19), 74.6% (95% CI 55.5–86.4%) for liposarcoma (*n* = 32), and 86.8% (95% CI 71.2–94.3%) for leiomyosarcoma (*n* = 38), indicating slightly better effectiveness for liposarcoma and leiomyosarcoma. The median OS in this study (11.7 months) was better than the gemcitabine/docetaxel combination but shorter than the OS with chemotherapy with either doxorubicin or epirubicin, trabectedin, or pazopanib. However, eribulin treatment seemed effective and may serve as an alternative treatment for these patients.

For myxofibrosarcoma, the median OS of trabectedin was 10.6 months (*n* = 3) in a retrospective study [40]. In another retrospective study of second-line gemcitabine-containing chemotherapy after doxorubicin treatment [43], the median OS was 11.4 months (*n* = 7). Although both studies included a small number of patients, the median OS was similar to the present study (11.1 months). Regarding malignant peripheral nerve sheath tumors, the median OS of gemcitabine/docetaxel combination in a second-line study was 13.0 months (*n* = 4) [39], and that of trabectedin in a retrospective observational study was 11.9 months (*n* = 4) [40]. The median OS of these drugs was longer than the median OS achieved in this study (3.3 months). The median OS for liposarcoma in this study was 20.8 months (*n* = 69), which was very favorable. As all patients treated with eribulin for malignant STSs were included in the analysis, all subtypes of liposarcoma (well-differentiated liposarcoma, myxoid liposarcoma, pleomorphic liposarcoma, and dedifferentiated liposarcoma) were included in the OS analysis. In patients with well-differentiated liposarcoma (*n* = 7), the median OS was not reached. Of those with liposarcoma, 10% were well-differentiated. It should be noted that the longer OS in patients with liposarcoma in this study may have been influenced by the results of patients with well-differentiated liposarcoma, which present less distant metastases.

We report a manageable safety profile for eribulin in the real-world setting. The present study revealed no differences in either the incidences of all Grade ADRs or Grade ≥3 ADRs between patients <65 and ≥65 years of age. The similar incidence of ADRs in patients ≥65 years and <65 years may be related to the fact that the proportion of patients who received a reduced initial dose of eribulin was higher in patients aged ≥65 years than those aged <65 years. The safety profile observed in the present study is in line with the known safety profile of eribulin, as the main ADRs were associated with myelosuppression [20, 22, 31]. Although not directly comparable to our study because we report ADRs while Schöffski et al. and Kawai et al. reported AEs, both trials reported a high incidence of Grade ≥3 or higher myelosuppression-related events (neutropenia, leukopenia, lymphopenia, and anemia) [20, 22]. The overall safety profiles between these two studies do not appear to be significantly different.

The proportion of patients who received a lower initial dose of eribulin (1.1 mg/m^2^) was highest among those aged ≥75 years (34.7%). This increased usage of a lower initial dose may explain why the incidence of Grade ≥3 ADRs in this patient population was similar to that of younger patients (<65 vs <75 years, and ≥65 vs <75 years). Together, our safety and dosage findings could indicate that a low initial dose of eribulin may allow for controlling ADRs. In the present study, the most common ADR except for myelosuppression was peripheral neuropathy (11.8%). The incidence in our study is not considered high compared with previous Japanese real-world studies of eribulin (Watanabe et al., 16.8% [44]). Regardless, we note that the incidence of peripheral neuropathy in patients treated with eribulin is lower than that reported for taxane drugs [45].

The present study had several limitations. First, this was an observational study and may have been prone to bias and/or confounding. Additionally, the timing of imaging evaluation was not defined in detail. Therefore, there were limitations in ascertaining progressive disease in the shortest time possible. Second, given that STS is a rare disease, it was not possible to collect a sufficient number of cases for some STS subtypes. Finally, the evaluation of OS in the present study was defined by a maximum follow-up of 2 years. However, longer follow-up may be necessary for some STS subtypes.

## Conclusion

Although this was an observational study with a small study population and the results should be interpreted with caution, eribulin showed effectiveness and a manageable safety profile for patients with STS, but not for some non-L-type subtypes in terms of effectiveness. As Japan is the only country where eribulin is approved for all subtypes of STS, it is in a unique position to provide real-world safety and effectiveness data of eribulin to treat STS, particularly for rare subtypes for which eribulin is not approved for treatment outside of Japan. Eribulin effectiveness data are crucial for Japanese patients with STS as the current treatment options are limited. The OS data presented here are valuable for both patients and treating physicians when deciding on appropriate treatment options for STS, including rare subtypes.

## Supplementary Information

**Additional file 1.**

## Data Availability

The datasets supporting the conclusions of the article are not available due to contractual restrictions with the institutions that collected the data.

## Abbreviations

ADR: Adverse drug reaction
AE: Adverse event
CI: Confidence interval
CR: Complete response
DCR: Disease control rate
ECOG PS: Eastern Cooperative Oncology Group performance status
ORR: Objective response rate
OS: Overall survival
PFS: Progression-free survival
PR: Partial response
SD: Stable disease
STS: Soft tissue sarcoma
TTF: Time-to-treatment failure
