# Supplementary material for: Safety and effectiveness of eribulin in Japanese patients with soft tissue sarcoma including rare subtypes: a post-marketing observational study
Source: BMC Cancer. 2022 May 11;22:528. doi: 10.1186/s12885-022-09527-y (PMC9092704; doi:10.1186/s12885-022-09527-y)
Supplement: Supplementary file 1 — Additional file 1. [file 12885_2022_9527_MOESM1_ESM.docx]

# Supplementary Information

**Additional file 1:** Best overall tumor response and time-to-treatment failure according to STS subtype in subtypes for which there were fewer than 5 patients

| **Soft tissue sarcoma subtype** |  | **Response rate, *n*** | | | | | | **ORR**  **(CR + PR)** | **DCR** | **TTF** | |
| --- | --- | --- | --- | --- | --- | --- | --- | --- | --- | --- | --- |
|  | ***n*** | **CR** | **PR** | **SD** | **SD**  **(≥11W)** | **PD** | **NE** | **(%)** | **(%)** | ***n*** | **median, months**  **(95% CI)** |
| Spindle cell sarcoma | 4 | 0 | 0 | 0 | 0 | 4 | 0 | 0.0 | 0.0 | 4 | 1.8 (0.5–2.3) |
| Epithelioid sarcoma | 3 | 0 | 0 | 2 | 1 | 1 | 0 | 0.0 | 66.7 | 3 | 2.6 (0.7– –) |
| Malignant solitary fibrous tumor | 3 | 0 | 0 | 0 | 0 | 3 | 0 | 0.0 | 0.0 | 3 | 2.8 (2.6–6.7) |
| Undifferentiated sarcoma | 2 | 0 | 0 | 1 | 0 | 1 | 0 | 0.0 | 50.0 | 2 | 1.7 (1.2–2.3) |
| Desmoplastic small round cell tumor | 2 | 0 | 0 | 1 | 0 | 1 | 0 | 0.0 | 50.0 | 2 | 3.5 (1.4–5.6) |
| Phyllodes tumor | 2 | 0 | 0 | 1 | 0 | 1 | 0 | 0.0 | 50.0 | 3 | 2.8 (0.8–3.7) |
| Undifferentiated round cell sarcoma | 1 | 0 | 1 | 0 | 0 | 0 | 0 | 100.0 | 100.0 | 1 | 4.2 (– – –) |
| Intimal sarcoma | 1 | 0 | 0 | 1 | 1 | 0 | 0 | 0.0 | 100.0 | 2 | 10.7 (1.7 – 19.7) |
| Extraskeletal myxoid chondrosarcoma | 1 | 0 | 0 | 1 | 1 | 0 | 0 | 0.0 | 100.0 | 1 | 8.0 (– – –) |
| Adenosarcoma | 1 | 0 | 0 | 1 | 1 | 0 | 0 | 0.0 | 100.0 | 1 | 8.3 (– – –) |
| Paraganglioma | 1 | 0 | 0 | 1 | 1 | 0 | 0 | 0.0 | 100.0 | 1 | 3.0 (– – –) |
| Uterine tumor resembling ovarian sex-cord tumor | 1 | 0 | 0 | 1 | 1 | 0 | 0 | 0.0 | 100.0 | 1 | 5.6 (– – –) |
| Pleomorphic spindle/epithelioid sarcoma | 1 | 0 | 0 | 1 | 0 | 0 | 0 | 0.0 | 100.0 | 1 | 0.7 (– – –) |
| Alveolar soft part sarcoma | 1 | 0 | 0 | 1 | 0 | 0 | 0 | 0.0 | 100.0 | 1 | 3.0 (– – –) |
| Sclerosing epithelioid fibrosarcoma | 1 | 0 | 0 | 1 | 0 | 0 | 0 | 0.0 | 100.0 | 1 | 1.7 (– – –) |
| Ewing’s sarcoma | 1 | 0 | 0 | 0 | 0 | 1 | 0 | 0.0 | 0.0 | 1 | 0.7 (– – –) |
| Mesenchymal chondrosarcoma | 1 | 0 | 0 | 0 | 0 | 1 | 0 | 0.0 | 0.0 | 1 | 2.6 (– – –) |
| Carcinosarcoma | 1 | 0 | 0 | 0 | 0 | 1 | 0 | 0.0 | 0.0 | 1 | 1.6 (– – –) |
| High grade fibrosarcoma | 1 | 0 | 0 | 0 | 0 | 1 | 0 | 0.0 | 0.0 | 1 | 0.7 (– – –) |
| Undifferentiated pleomorphic sarcoma | 1 | 0 | 0 | 0 | 0 | 1 | 0 | 0.0 | 0.0 | 1 | 0.5 (– – –) |
| Carcinosarcoma uterus | 1 | 0 | 0 | 0 | 0 | 1 | 0 | 0.0 | 0.0 | 1 | 1.6 (– – –) |
| Aortic hemangioendothelial sarcoma | 1 | 0 | 0 | 0 | 0 | 1 | 0 | 0.0 | 0.0 | 1 | 0.7 (– – –) |
| Chondrosarcoma | 1 | 0 | 0 | 0 | 0 | 1 | 0 | 0.0 | 0.0 | 1 | 1.4 (– – –) |
| Breast malignant phyllodes tumor | 1 | 0 | 0 | 0 | 0 | 1 | 0 | 0.0 | 0.0 | 1 | 1.9 (– – –) |
| Dermatofibrosarcoma protuberans | 1 | 0 | 0 | 0 | 0 | 0 | 1 | 0.0 | 0.0 | 1 | 1.4 (– – –) |
| Pleomorphic sarcoma | – | – | – | – | – | – | – | – | – | 1 | 0.5 (– – –) |
| Endocardial sarcoma | – | – | – | – | – | – | – | – | – | 1 | 0.7 (– – –) |
| Ovarian carcinosarcoma | – | – | – | – | – | – | – | – | – | 1 | 0.5 (– – –) |

*CI* confidence interval, *CR* complete response, *DCR* disease control rate, *NE* not evaluable, *PD* progressive disease, *PR* partial response, *SD* stable disease, *STS* soft tissue sarcoma, *TTF* time-to-treatment failure, *W* weeks
